# Toward a paradox-oriented paradigm of knowing: the form theory of observation and quality in empirical sciences

**DOI:** 10.3389/fsoc.2026.1758071

**Published:** 2026-06-04

**Authors:** Mario S. Staller, Swen Koerner

**Affiliations:** 1 Department of Police, University for Police and Public Administration North Rhine-Westphalia, Cologne, Germany; 2 Department of Training Pedagogy and Martial Research, German Sports University Cologne, Cologne, Germany

**Keywords:** empirical science, epistemic quality, form theory, paradox, reflexivity, scientific rigor, systems theory, treatment-prevalence paradox

## Abstract

We develop a form-theoretical account of knowing in empirically oriented sciences and argue for a paradox-oriented understanding of epistemic quality. Drawing on Spencer-Brown and Luhmann, we start from the idea that observation always operates by drawing a distinction and indicating one side. What appears as an object or “phenomenon” is thus the marked side of a form; its unmarked side persists as horizon. This implies an origin paradox of knowing: any knowing system can only operate by means of distinctions whose own contingency cannot be observed in the very act of use. We reconstruct how this paradox is functionally invisibilized and proliferates within science understood as a social system operating under the guiding distinction true/not-true. Programmes (methods, designs, standards), organizations and memory structures (journals, universities, reviews) distribute the paradox across three dimensions of scientific communication: in the factual dimension, as object formats, evidential hierarchies and growing residual categories; in the social dimension, as roles, communities and responsibility attributions; and in the temporal dimension, as replication cycles, escalation programmes and crisis semantics. This stabilizes an implicit quality paradigm that equates quality with coherence, consensus and cumulative stability. As an alternative, we propose a paradox-oriented quality concept: quality in this perspective lies in how a system addresses the constitutive paradox of its own observation without blocking its autopoiesis. We conceptualize quality as a calibrated rhythm of invisibilizing and briefly exposing the form of observation, increasing both robustness and the range of next possible operations. While developed with reference to empirical science, the argument generalizes to any knowing system.

## 1 Introduction

The following reflections on knowing in knowing systems (such as science) are not new. They have been extensively developed in systems-theoretical and form-theoretical work (Baecker, 1999, 2021; Luhmann, 1996, 1999b,a; Spencer-Brown, 1979). The radical consequences of these reflections, however, seem to be perceived only marginally

in scientific discourse. This applies especially to the empirical sciences: the operation of observation brings its object into form, and it does so by distinctions that are at once the condition and the limit of what can be observed (Körner, 2014; Nassehi and Saake, 2002; Staller and Koerner, 2022). With this paper, we aim to increase the reflexive potential particularly for those sciences that operate under the empirical paradigm.

Especially in the social, behavioral and life sciences—for example in psychology, psychiatry, data-intensive social research and application-oriented fields—this empirical paradigm has come under pressure in recent years (e.g., Hillary and Medaglia, 2020; Moody et al., 2022; Murphy et al., 2025; Tackett et al., 2019). At first, the empirical data themselves are disturbing: effects fail to replicate (Maxwell et al., 2015), findings show substantial dispersion (Breznau et al., 2022), prevalence trends behave in contradictory ways (Ezawa et al., 2024; Ormel et al., 2022). This empirical unrest is semantically condensed as a “replication crisis” or “crisis of confidence” in the empirical sciences and generates pressure to act. A central response has been the reform bundle of the open science movement (Renkewitz and Heene, 2019): preregistration, registered reports, transparency norms, standardization, and replication consortia are intended to restore coherence and reliability through a “more of method.”

Under these tightened conditions, further irritations become visible. In the epidemiology of mental disorders, for instance, the treatment–prevalence paradox is discussed (Batstra and Timimi, 2024; Ezawa et al., 2024; Ormel et al., 2022)—not despite, but precisely because of expanding diagnostic, screening and service structures: improved treatment and increased awareness seem to coincide with stable or rising prevalence rates. In metascientific many-analysts studies, numerous research teams work with the same data and the same hypothesis, under explicit orientation toward scientific method, high ethical standards and reproducibility requirements; nevertheless, the results range from clear confirmation to clear rejection of the hypothesis (Breznau et al., 2022). The empirical variance does not disappear; rather, it becomes visible precisely under open-science-oriented conditions. The crisis semantics shifts: alongside the replication crisis, a theory crisis emerges (Green, 2021; Oberauer and Lewandowsky, 2019); it appears that the problem lies not only in “the method” but in the epistemic foundations themselves (Mayrhofer et al., 2024; Uher, 2025). While parts of the (empirical) community explicitly reflect this shift, others hold on to the previous mode of problem processing: the problem continues to be treated as an empirical deficit that can be solved by better interventions and the call for more empirical research, which synthesizes into the mantra “more research is needed.”

The proposal of this paper is to read this pattern in form- and systems-theoretical terms: the more strongly (empirical) science attempts to control its object through methodological observation, the more clearly what is observed eludes such control. We understand this phenomenon as an expression of an origin paradox of knowing systems. Every knowing system can only observe by using a distinction (such as inside/outside or true/untrue). Yet this very distinction cannot be co-observed at the moment of its use—it remains the structural blind spot of observation. When this difference is held functionally invisible in order to make decisions possible, it shifts and reappears elsewhere in observation. Building on this diagnosis, we develop reflections on a concept of quality in knowing systems. We propose to assess quality by asking whether a knowing system addresses the constitutive paradox of its own observation in such a way that its autopoiesis—understood as the ongoing self-production of the system through its own operations—becomes more robust vis-à-vis irritations, without leaving the guiding distinction within which it operates (Luhmann, 1996). Our argument is primarily descriptive-analytical, insofar as it reconstructs how scientific quality is organized through forms of observation. It is also normatively relevant in a conditional sense: if science aims to increase rigor and robustness without suppressing its own constitutive blind spots, then quality may be assessed by how it organizes the temporary visibility of observational forms.

We unfold our thesis in six steps. Although we develop our argument with a focus on empirical sciences, the underlying diagnosis is framed more generally: any knowing system that operates through observation and a binary code faces the same origin paradox and must find ways to keep it functionally invisible without blocking its own operations. First (Section 2), we introduce a form theory of observation as our starting point: to observe is to distinguish and to indicate; what appears as an observation is the marked side of a form. We then clarify (Section 3) the self-referential relations of knowing and the inescapable paradox involved: the system's own distinction can only become visible as a short-lived re-entry, that must be rendered unobservable in order to be functional. Section 4 describes science as a social system with a truth code (true/not-true), programmes and a traditional quality programme that reduces selection pressure and functionally invisibilizes paradoxes. We show how the origin paradox proliferates along the factual, social and temporal dimensions and illustrate this, among other things, with reference to the epidemiology of mental disorders, many-analysts findings and the open science movement. Section 5 sketches a programme shift from a coherence-oriented to a paradox-sensitive quality paradigm, formulated as a conditional internal claim for the robustness of autopoiesis. Section 6 bends the proposal back onto itself: the paradox of paradox as reflexivity of form. We conclude with a brief epilog (Section 7), which explains why this perspective, from a systems-theoretical point of view, has so far only selectively found resonance in prevailing understandings of science—and may continue to do so.

## 2 The form theory of observation as a basis for a theory of knowing

Niklas Luhmann's systems theory is known for its complexity, its idiosyncratic language, but also for its internal coherence and its view of the world in terms of system/environment distinctions (Lee, 2012). The theory conceptualizes systems (social as well as psychic) as autopoietic, self-referential systems (Luhmann, 1986). In developing his systems theory, Luhmann presupposes a fundamental distinction: the difference between system and environment. His further theoretical edifice is built upon this guiding distinction—theoretically justified but ultimately set. Luhmann links this setting to the question of the beginning of knowing: every human cognition of human cognition is itself again human cognition. To address this self-referential circle, he turns to Spencer-Brown's (1979) Laws of Form. Its form calculus offers a way not to bypass the necessary self-referentiality of observation, but to make it productive for epistemic purposes. Luhmann understands the idea of form developed there—form as the unity of a distinction—as a basic theoretical figure underlying his systems theory, to which he attributes the potential to be generalized far beyond systems theory itself (Luhmann, 2004).

In what follows, we first outline the core elements of this form-theoretical account of observation, which then provides the basis for our systems-theoretical account of knowing: in order to “know” something, there must be observation. And to observe means to draw a distinction and to indicate one side. With the indication, an object does not arise as something given, but as the marked side of a form whose other side remains unmarked—what (Spencer-Brown 1979) describes as the unmarked state within an unmarked space—and accompanies the operation as a horizon. What appears as “the phenomenon” owes its visibility to the difference against which it is marked. From this perspective, “it” is nothing ontological, but an effect of form.

Radical consequences follow from this formal insight. There is no knowledge independent of, and detached from, the observations of an observer. Nor is it neutral. As an operation of an observer, every observation is value-laden: “It is therefore the evaluation by the observer that determines where a distinction is drawn and which contents are indicated” (Simon, 1993; p. 59; see also Spencer-Brown, 1979).

Observation thus always actualises only one possibility among other possibilities. Actuality is surrounded by a horizon of meaning that carries alternatives along without actualising them at the same time (Luhmann, 1990b). From this perspective, the form of observation becomes the guiding parameter of observation itself: it selects what can appear; it structures which subsequent operations become plausible; it determines which deviations become visible as deviations. Changes in observational practice—new criteria, different measurement practices, altered categories—are therefore primarily changes of form: shifts in the distinctions by which observation brings the observable into being. The form theory of observation thus makes clear that it is not contents that carry observation, but the differences within which contents can appear as contents at all.

Based on these considerations, we now turn back to the self-referential circle of knowing and the associated origin paradox of knowledge.

## 3 Self-referential relations and the unavoidable paradox of knowing

From a systems-theoretical perspective, “knowing” consists of self-referential processes. A knowing system—for example a “subject,” a psychic system or a social system such as science—is always part of the world it seeks to know. Even the question of whether the system exists or not—the question of being or not-being—must be answered with the system's own means of knowing. Only its own operations are available for the process of knowing.

Knowing begins with the observation of an operation. Observations lead to knowing insofar as reusable results are stabilized within the knowing system (Luhmann, 2005), that is, insofar as further observations become more or less likely. In the operation of observing, a paradox becomes visible. It becomes observable when a system observes the conditions of possibility of its own knowing. In this sense, the paradox is constitutive of observation and, consequently, of knowing. Observation is only possible as an operation; otherwise, it could not occur. The distinction between operation and observation is itself only possible as observation, since observing is indicating within a distinction. If an operation is to be observed, it must therefore be distinguished. This leads to a circle of mutual presuppositions which can only arise on the condition that the circle itself does not appear while it is circling. It is “a tautology which in turn conceals the paradox of observing” (Luhmann, 1995c; p. 21).

It is a unity that exists as unity only in and through difference.

If we become entangled in this tautology/paradox, there is no way out. Things are simply what they are, and further observations—beyond the insight that we could not observe—would be blocked. The unity of the difference between operation and observation cannot be dissolved (Luhmann, 1995a). For operations to continue nonetheless and for a system to be able to continue itself through its own operations (autopoiesis), formats must be found that allow the unobservable to be observed. The system must protect itself against the blockage of its autopoiesis, and this is precisely the function of paradoxes. “Very roughly, they serve to separate operations and observations. […] Paradoxes allow operations to occur, but they block observations. One can certainly think paradoxically and also communicate paradoxically. […]. But if one wants to observe, that is, to distinguish and indicate what is meant, then this (and only this!) operation is blocked. It oscillates between the two sides it intends to distinguish and cannot decide on which side subsequent operations should be connected” (Luhmann, 1995b; p. 34).

This origin paradox of knowing—the unity of the difference between observation and operation—must be dealt with in one way or another, regardless of which epistemological logic one prefers. Otherwise, nothing follows. “All epistemology has to begin with the resolution of a paradox” (Luhmann, 2005, p. 36).

The form-theoretical considerations outlined above make the paradox tractable in that they carry it along productively. At the operational level, one must first begin by drawing a distinction (“draw a distinction”); through the subsequent step in Spencer-Brown's (1979) calculus—the introduction of the form into the form (the so-called re-entry) —it becomes clear that observation coincides with the first distinction. The operation of distinguishing and the act of observing are identical as a unity of difference and together constitute the constitutive paradox of the form of knowing. Luhmann himself points, for the productive handling of this paradox, to the theory of autopoietic systems, which, in recursive operations, produce and reproduce a network of such operations and thereby create the very conditions of possibility for this reproduction. In such systems there is no operation without references to other operations of the same system. Correspondingly, Luhmann proposes to resolve this tautology/paradox by introducing, as observer, the first distinction^1^ between system and environment. The observer may remain invisible as observer but can still allow an autological inference to themself. For if this is to be the first distinction, then the observer must also (in case someone wishes to observe the observer) be a system. This first distinction de-ontologizes the world. If one starts from it, the distinction between being and non-being can only claim secondary status; it now depends on a system-in-the-world what can function for it as being and as non-being. The scope of this first distinction can hardly be overstated (Luhmann, 1995c).

Empirical sciences particularly often proceed from ontological realities (see, e.g., the empirical strand. of psychology; Mayrhofer et al., 2024; Staller and Koerner, 2025). Things “are” and only need to be correctly grasped—with epistemological tools. From a systems-theoretical perspective, the frequently encountered separation of subject and object can be read as a successful attempt to deal with the inherent “paradox of self-reference” (Luhmann, 1995a, p. 106). Whoever wants unity must generate difference. In doing so, however, the constitutive paradox is functionally forgotten. The paradox does not disappear—it is merely displaced. It proliferates. In the following, we trace how these displacements become visible in the scientific system as specific patterns of proliferation.

## 4 Proliferation of the origin paradox of knowing in the (empirical) sciences

Science can be described as a social system whose elements are communications that connect to one another by reference to a binary code: true/not-true (Luhmann, 1990a). The code functions as a selection schema that determines which communications can count as scientifically connectable; linked to it is the symbolically generalized medium of truth, which increases the chances of acceptance when communications are marked as true. Both sides of the code are necessary: the preferred side “true” serves as a target structure, the rejection side “not-true” as a starting point for further testing. In this way, decisions under conditions of uncertainty become possible without leaving the code itself.

For the code to be applicable in actual operations, programmes are required. Programmes are historically variable arrangements of rules, methods, quality criteria and formats of presentation that specify under which conditions a communication can count as “true.” They range from designs, measurement practices and inference regimes to replication formats, reporting standards, evidence hierarchies and transparency requirements. Taken together, they form a quality programme that organizes the application of the code without substituting it: quality appears not as a property of things, but as a programme form that reduces selection pressure and makes the operation of the truth code workable.

Organizational structures—universities, institutes, journals, professional societies—embed these programmes in decisions. Acceptance or rejection of a manuscript, allocation or non-allocation of funds, appointment or non-appointment to a position condense into organizational nodes at which the code is applied under specific programmes (Luhmann, 1990a). The publication system assumes the memory function by storing past selections and making them available for present operations of connection in the form of literature, citations, reviews and meta-analyses. In this interlacing of code, programmes, organizations and memory, the scientific programme of knowing is realized: it aims to translate uncertainty into decidable selections of truth—and must, to do so, keep its own constitutive invisibilities functionally intact.

This is precisely where our analysis begins. The origin paradox of knowing—that every observation is only possible in the mode of a drawn distinction which carries its own contingency along—cannot be kept permanently visible in ongoing operations without blocking the system's functioning. It is invisibilised via programmes, organizations and quality semantics, but remains effective. In what follows, we show how this origin paradox proliferates along the three dimensions of meaning in scientific communication: in the factual dimension, where it is sedimented in object and evidence formats; in the social dimension, where it migrates into roles, community structures and attributions of responsibility; and in the temporal dimension, where it is outsourced into replication cycles, escalation programmes and crisis semantics.

### 4.1 Factual dimension: proliferation in the “what” of observation

In the factual dimension, proliferation appears where the basic difference of the scientific code (true/false) is translated into concrete programme distinctions that structure the “what” of observation. Invisibilization of the origin paradox occurs through semantic condensation and object formatting. From the formal difference true/false, manageable oppositions are derived: significant/non-significant, robust/non-robust, high-quality/low-quality, evidence-based/non-evidence-based. Thresholds such as *p* < 0.05, effect size categories, quality labels or evidence grades function as selection hinges at which the original undecidability is translated into seemingly factual criteria (see, for example, Luhmann's notion of “statistical depression”; Luhmann, 2000).

Within this framework, we can observe that programmes are continually being condensed. Procedures, checklists, and catalogs of criteria become more extensive, method sections grow, reporting standards differentiate. The increase in formatted distinctions raises the density of factual specifications without proportionally increasing decision clarity. The more distinctions are introduced, the more clearly a second effect emerges: the number of residual cases—exceptions and gray zones—increases. Proliferation appears here as a proliferation of differences that ever more finely structure the “what” of observation while simultaneously generating new indeterminacies.

At the same time, meta-formats increasingly stabilize alongside primary studies: systematic reviews, meta-analyses, method comparisons or reporting guidelines that operate at the level of the observed “object.” They claim to order the heterogeneous material “beneath” them, but in turn generate divergences: different inclusion criteria, evaluation regimes and model assumptions lead to controversies over which meta-perspective may count as the observational format for “the evidence.” The origin paradox thus migrates into the selection of formats that are used as factual framings for what is to count as the “state of findings.”

Where programmes and meta-formats reach their limits, incompatibilities between them are collected in residual categories: heterogeneity, publication bias, model assumptions, setting effects. Such residues frame the results without taking over the decision itself. It is precisely in these residual classes that the previously invisibilised form returns as a problem of factual validity—it becomes visible that the order of the material does not stem from the material itself, but from the form of observation.

The following examples illustrate this pattern of movement. For example, in psychology, the programme distinction significant/non-significant replaces the code true/false in the actual conduct of observation. The choice and handling of the threshold, for instance *p* < 0.05, shifts the paradox into the factual format of a cut-off value: HARKing, *p*-hacking, fishing (Andrade, 2021; Earp and Trafimow, 2015; Lishner, 2021) or other practices that are now termed “questionable research practices” (John et al., 2011; Uher et al., 2025) appear as attempts to produce follow-up decisions within this format. Once the use of thresholds is problematized, the tension shifts into meta-factual dimensions: “new statistics” (Cumming, 2014), preregistration (Nosek et al., 2018), large-scale collaborations (Moshontz et al., 2018), and other interventions (Nosek et al., 2021) are supposed to create clarity, but themselves generate new differences. Residual categories then arise such as context dependence, lab effects or measurement invariance, in which the original problem—the visibility of form—reappears as heterogeneity of effects. Replications then do not primarily make “reality” visible, but divergences in the factual form of observation—for example, with regard to sample construction, operationalisation or analysis regimes. The paradox appears as a difference of difference: identically worded hypotheses become differently selectable as true/false under differently formatted observations.

In biomedical and clinical-epidemiological research, evidence hierarchies structure the factual dimension by ordering study designs according to their proximity to a gold standard such as the randomized controlled trial (Djulbegovic and Guyatt, 2017). The basic difference is built into programme logics intended to maximize internal validity. The unmarked counter-horizon migrates into questions of transferability—population differences, surrogate endpoints, setting effects—and into procedures for aggregation such as meta-analyses or network meta-analyses. At these factual coupling points, residues accumulate in the form of heterogeneity measures, publication bias and small-study effects, or model choice decisions (Egger et al., 1997; Huedo-Medina et al., 2006). Against this background, the treatment–prevalence paradox can be read as a form effect: prevalence rates respond sensitively to the method of observation (Levis et al., 2020). What appears as “more” or “less” mental disorder depends crucially on how cases are counted, how symptoms are aggregated, and how screening procedures are deployed. Recent work on the treatment–prevalence paradox in depression and anxiety illustrates this tension between expanding treatment and relatively stable or increasing prevalence estimates (Ezawa et al., 2024; Ormel et al., 2022). The origin paradox manifests as a tension between an apparently stable object (“prevalence”) and the form of its observation.

In data-dense fields that work with complex analysis pipelines, the origin paradox likewise first shifts into the factual dimension. As more and more variables are measured and models become more flexible, the number of possible model families and performance criteria grows. Each additional metric—for example overall accuracy, different loss functions, or indices for fairness and generalisability—opens up a new vantage point from which the same data can be judged (Hand, 2012; Lipton, 2018). Many-analysts studies render this dynamic visible: numerous teams work with the same data and the same hypothesis but rely on differently formatted decision trees, model classes and analysis routines (Botvinik-Nezer et al., 2020; Breznau et al., 2022; Silberzahn et al., 2017). The choice of a metric and a model provide local relief by clarifying, for the moment, what is to count as “better”; globally, new incompatibilities emerge between metrics, models and interpretations. The paradox is distributed across an increasing number of factual formats, without any one of them being able to definitively displace the others.

### 4.2 Social dimension: proliferation in the “who” of observation

In the social dimension, proliferation appears where the origin paradox is translated into attributions of observation, competence, and responsibility. Invisibilization occurs by describing observations not as effects of a contingent form, but as achievements of particular roles, committees, or research communities. Instead of asking how observation is conducted, the focus shifts to who is doing “clean,” “robust,” or “rigorous” research. The paradox thus migrates into the relation between alter and ego, into structures of recognition, reputation and contestation in which scientific claims are stabilized or marginalized (Latour and Woolgar, 1979).

Peer review regimes form a central figure of this shift. Peer review externalizes undecidabilities to a social review mechanism. “Robustness” and “rigor” emerge as semantics that cover the basic difference: rejection or acceptance is attributed to the judgment of a peer community, not to the effect of a specific form of observation (Bornmann, 2011; Lee et al., 2013; Waltman et al., 2023). When reviews conflict, the tension is displaced into editorial decisions, additional reviews or special publication formats that formally process divergences. The underlying question of form—which distinction carries an observation—remains largely untouched.

In parallel, methodological schools, theoretical camps and disciplinary sections emerge that stabilize different expectation spaces. Within each of these spaces, a particular observational form counts as state of the art, while other formats appear marginal, deficient or simply not connectable. Proliferation shows up as an increasing number of such communities, each cultivating its own quality and evidence semantics (Kuhn, 1962). The origin paradox appears here as a persistent dissensus between camps that cannot simply be resolved by additional data, but only by shifts in structures of recognition—hence the recurrent appeal to interdisciplinarity as a remedy (see, e.g., Cleeremans et al., 2025). This social negotiation of form is not symmetrical. Power asymmetries influence which invisibilizations are accepted as legitimate quality and which are marked as bias, deficiency or misconduct. Senior researchers, dominant methodological schools, prestigious journals and well-resourced institutions can stabilize their observational forms more easily as “state of the art,” whereas marginal, junior or geographically peripheral actors may have to make their forms explicit under stronger justificatory pressure. From a form-theoretical perspective, power plays a central role in the proliferation of the paradox of observation in the social dimension. It participates in distributing whose blind spots can remain invisible and whose must become accountable (see, e.g., Abo-Zena et al., 2022; Awad et al., 2025).

Added to this are semantics of professionalism and responsibility that translate the problem of observational form into moral or normative demands. Quality campaigns, ethics guidelines, catalogs of good scientific practice and reform programmes address uncertainty in the form of expectations directed at researchers. “Misconduct,” “questionable research practices,” or “bias” are attributed to individuals, teams or institutions (Bouter, 2024; Fanelli, 2009; John et al., 2011). The question of how the form of observation produces undecidability appears, from this perspective, as a question of right or wrong behavior. The paradox of observation is thus processed on the social dimension by transforming uncertainties into attributions of responsibility and blame.

The fields already sketched can be read as social proliferation. Replication consortia, multi-center studies, many-analysts projects and open science initiatives establish new roles and committees in which it is decided which observational form may count as trustworthy: replication labs, data stewards, meta-science, method colleges. Divergent results are processed not only factually, but also socially—as differences that are discussed and coordinated among labs, networks, and consortia over which analyses, models, and interpretations should be trusted (Klein et al., 2014; Silberzahn et al., 2017). Even where authors themselves attribute remaining variation primarily to properties of the effects under study (Klein et al., 2014), the community-level handling of disagreement takes the form of social negotiation over which observational formats should guide future work. Where forms are not compatible, typical residual categories of the social dimension arise: controversial evidence base, lack of consensus in the community, further research needed. Such formulas relieve expectations without addressing the core of form theory—namely that all participants observe on the basis of an invisibilised origin paradox whose proliferation produces precisely the dissensus they then attempt to process socially.

### 4.3 Temporal dimension: proliferation in the “when” of observation

In the temporal dimension, the origin paradox shifts into programmes that regulate the duration, provisionality and revisability of knowledge. Invisibilization occurs by not resolving undecidability but postponing it: as a promise of future clarification, as a routine of cyclical review, or as a sequence of codified revision steps. Knowledge appears as a historically stratified sequence of states—initial evidence, robust evidence base, meta-analytically confirmed, obsolete—while each stage generates new demands for revision. Proliferation in the temporal dimension thus takes the form of a densification of repetition and escalation programmes.

Replication and audit cycles are a primary, salient form of this shift. Replications, audits and review loops outsource undecidability into recurring temporal structures. Decisions are taken under reservation, as provisional evidence, and secured by programmes of repetition. Each replication carried out creates additional points of comparison at which divergences become visible, as the large-scale replication efforts in psychology have demonstrated (Open Science Collaboration, 2015; Shrout and Rodgers, 2018). The origin paradox migrates into the difference between expected stability over time and empirically experienced variation. What is intended as a safeguard generates new occasions at which the question of form breaks open again.

In data-dense fields, models explicitly appear as provisional and updatable. Data cleaning, feature selection, hyperparameter tuning and model comparison are organized as temporal sequences in which each iteration provides local relief by allowing a model status to be accepted provisionally (Bouthillier et al., 2019). Globally, however, new sites of selection emerge: stopping rules, validation protocols and update cycles must be specified and carry undecidability forward. Progress materializes as a chain of programme decisions whose open ends transport the undecidable into the next round. The paradox here appears as a structural impossibility of marking a final point of decision.

Crisis semantics and escalation programmes add another layer to this dynamic. Replication crisis, crisis of trust, methods crisis or evidence crisis are temporal semantics that translate the origin paradox into dramatized diagnoses of the present (Earp and Trafimow, 2015; Sanbonmatsu et al., 2025; Tackett et al., 2019). Reform demands—more data, larger studies, stricter thresholds, denser protocols—are programmes of escalation oriented toward future improvement. The present is systematically placed under the obligation to correct the errors of the past and to realize a not-yet-achieved future. The paradox proliferates insofar as the very uncertainty that invisibilization was meant to relieve is built into the temporal structure of knowledge as a permanent demand for revision (Kuhn, 1962).

The treatment–prevalence paradox can again be read from this temporal perspective. Diagnostic and therapeutic programmes are historically expanded; prevalence series appear as time series on which “progress” is supposed to become readable. If rates nevertheless remain stable or increase, the paradox shifts into the difference between the expectation of cumulative improvement and the empirically observed development (Ezawa et al., 2024; Ormel et al., 2022). Similarly, many-analysts studies mark a point at which the system demands an interim balance of itself: under conditions that are supposed to promise “maximum reproducibility,” divergences become visible that simultaneously justify and perpetuate the call for “more research is needed” (Breznau et al., 2022). In psychology, this temporal vector can be observed particularly clearly: replication projects, preregistration initiatives and registered reports organize research processes along explicit time axes—before and after registration, before and after replication (Nosek et al., 2018; Open Science Collaboration, 2015). In all these cases, the core of form theory remains intact: every decision relieves the present but generates additional pressure for revision in the future and shifts the origin paradox into the expectation of later clarification.

### 4.4 Proliferation and the implicit quality concept of science

The manifestations in the factual, social and temporal dimensions show proliferation not as an exception, but as the normal mode of relief. The origin paradox is kept invisible so that observation remains capable of further connection; it returns at the coupling points between factual codes and programmes, between social attributions and roles, between temporal routines and escalation programmes. What becomes observable are condensations without proportional decision clarity, proliferations of meta-formats, growing residual classes and crisis semantics that address purity—in the sense of a return to the “proper” —or escalation—in the sense of more data, stricter thresholds, denser protocols.

Beyond the dimensions sketched here, the origin paradox also proliferates in the self-descriptions of disciplines, in the coupling to other function systems, and in symbolically generalized communication media such as truth, which are supposed to guarantee stability under uncertainty. In all these cases, proliferation proves to be a movement form of the paradox: it travels along its own relief devices, distributes itself across the coordinates of meaning, and becomes noticeable precisely where invisibilization has been most effective.

In this way, an implicit quality concept becomes visible that in fact guides empirical sciences. In the factual dimension, quality appears as coherence of objects and evidence formats: contradictions are to be minimized, findings synthesized, heterogeneities absorbed into residual categories. In the social dimension, quality is coded as capacity for consensus: peer review, referee reports, community standards and professionalism semantics are supposed to bring divergent observational forms to a workable common denominator. In the temporal dimension, finally, quality is equated with stability and capacity for escalation: robust effects, replicable findings, cumulative evidence and reform programmes that promise “more” and “better.” This quality concept fulfills its function by closing differences and thereby enabling decision under uncertainty. At the same time, the analysis of proliferation shows that the constitutive paradox of knowing does not thereby disappear, but migrates into factual, social. and temporal dimensions. The blind spot may be that quality is treated primarily as the outcome of achieved coherence, while the conditions of its production—the form along which truth is selected—remain invisible in the actual operation. This is where we take up the argument in the following section: we propose to orient quality not toward the erasure of difference, but toward the addressed visibility of form.

## 5 Quality in science: from coherence to paradox

The foregoing analysis has shown that empirical sciences stabilize an implicit concept of quality: quality is attributed wherever differences are closed, contradictions are pacified, and unstable observations are transformed into stable narratives. Quality thus appears as a by-product of coherence-oriented relief—in the factual dimension as smoothing contradictory findings, in the social dimension as the capacity of communities to reach consensus, and in the temporal dimension as stability, replicability, and capacity for empirical escalation. For example, the treatment-prevalence paradox points toward a problem within this logic: it irritates precisely where coherence is expected and is therefore read primarily as a deficit rather than as signal of form.

The following proposal does not start from this coherence semantics, but from the form-theoretical condition of knowing. If every scientific observation presupposes that a system draws a distinction and keeps its contingency invisible in the act of operating, then quality is not, in the first instance, a property of results but a way of dealing with this constitutive paradox. Quality may here denote those practices in which a system addresses its own form—the unity of the difference true/not-true—in such a way that both the range of next possible operations and robustness increase, without the operation of the system collapsing. The question shifts: no longer “How coherent are the results?,” but “How well does the system manage to make its own form selectively visible and still decide?”

A paradox-sensitive quality programme of this kind would not be an add-on to existing methods, but a recoding of what counts as “good” execution of scientific operations. Quality would then lie in calibrating a corridor of visibility: the basic distinction may briefly and selectively surface at certain points in research practice—for example when criteria for criteria, validations of validations, or aggregations of aggregations are reflected—and must then again be invisibilised to the extent that decisions are not indefinitely postponed. Quality would thus be neither permanent transparency nor complete concealment, but a structurally generated rhythm of exposure and relief. Operationally, a paradox-oriented quality practice can be identified where a research process contains institutionally specified moments in which the form of observation is made explicit, without turning this reflexivity into permanent suspension of decision. We propose three indicators as relevant: first, whether the distinctions that format the object are named, for instance diagnostic thresholds, model choices, inclusion criteria or validity assumptions; second, whether alternative distinctions are made comparable rather than immediately treated as error; and third, whether the process contains a return mechanism that translates this exposure back into decidable next operations. Calibration, in this sense, would be a temporal and organizational arrangement: exposure is productive when it increases the range of possible follow-up operations; it becomes excessive when it prevents any further selection. Although we articulate this proposal in the mode of a quality semantic for empirical sciences, the underlying idea is not restricted to this function system: wherever a system's operations are bound to observation under a code, quality can be understood as the way in which the system addresses its constitutive paradox such that robustness and possibilities of further connection increase.

In the factual dimension, this means that phenomena such as heterogeneity, model conflicts and context dependencies are not treated primarily as disturbances of a coherent body of knowledge. They can be read as indicators of form: they mark points at which different distinctions are at work, where operationalisations, thresholds or aggregation rules diverge. A paradox-sensitive understanding of quality would not seek to neutralize such form signals as quickly as possible, but would build them into reports, models and syntheses in such a way that it remains visible—and open for further operations—which assumptions about form produce which differences.

Empirical discussions of the treatment–prevalence paradox in depression have shown that expanded diagnostic and therapeutic programmes have not been accompanied by corresponding declines in prevalence estimates (Ezawa et al., 2024; Ormel et al., 2022). From a form-theoretical perspective, such patterns can be read as indications of how strongly prevalence estimates are coupled to diagnostic formats, cut-off points and survey designs, and how these couplings are reflected in stable or rising rates. Quality, on this view, would be achieved when factual products are constructed in such a way that the form of observation remains readable, without thereby suspending every decision. The aim would be a structured visibility of the distinctions that organize evidence, so that decisions about “what the data show” can be made in awareness of the forms that make these data possible.

In the social dimension, the concept of quality shifts from agreement on results to the capacity of form to re-enter social structures. Peer review, editorial decisions, guideline processes and the positions of professional associations would be of higher quality where they not only issue judgements on rigor, correctness, or relevance, but explicitly indicate which distinctions underpin their evaluations. Divergent results could then initially be treated as expressions of different observational forms—different ways of formatting samples, different assumptions about normality, different concepts of error—before they are processed in moral, reputational or hierarchical terms. Consensus would still have a function, but would count as temporary prioritization of certain forms, not as final erasure of difference. Social quality would mean organizing procedures and roles in such a way that questions about form—“Exactly what is being distinguished here, and how?”—are institutionally provided for, rather than being mere occasional disruptions of routine operations. Many-analysts studies, in this perspective, acquire a different status: the variance of results is not the deficit, but the material in which different form decisions by different teams become comparatively visible (Breznau et al., 2022); qualitatively, the point would be not to individualize this variance, but to process it as structured difference between observational forms. Many-analysts studies provide a concise example of this rhythm. In the routinized phase, each team must invisibilize its own form sufficiently to produce an analysis: variables are selected, models specified, robustness checks chosen, and a result is reported. In the exposure phase, the study design compares these analyses side by side and thereby makes visible that “the same” hypothesis and “the same” data become different objects under different analytical distinctions. In the return phase, the variance is not simply dissolved into error or averaged away, but can be used to specify which observational forms generate which results. The quality gain would lie precisely in this sequence: local invisibilization enables decisions, comparative exposure makes form visible, and subsequent interpretation restores connectability.

In the temporal dimension, finally, a paradox-sensitive quality programme would no longer treat replications, audits, re-analyses and model updates merely as means to stabilize an already established body of findings. They would become structured occasions on which the system confronts itself with its own form again. Under these conditions, a replication initiative would not only be successful when it confirms effects, but also when it brings to light differences that can be traced back to changed observational formats—and when these differences are explicitly processed in subsequent programmes. Temporal quality would consist in coupling phases of form exposure and phases of form forgetfulness in such a way that neither side becomes absolute: neither a permanent crisis in which everything is up for negotiation, nor a permanent rhetoric of stability in which every irritation is discounted as an outlier. Open-science programmes would then not primarily be read as escalation programmes of “more data, more transparency,” but as infrastructures that enable targeted re-entries of form (for example via preregistration, data sharing, many-analysts designs)—of higher quality where they do not merely accumulate additional procedures, but open time windows in which observational forms can become visible and revisable before operations return to routinized execution.

In all three dimensions of meaning, the issue is therefore less one of additional demands than of a different reading of what is already happening. The proliferation phenomena described—densification of programmes, proliferation of meta-formats, growth of residual classes and crisis scenarios—show that science is already grappling with its own paradox but largely interprets it as a deficit: as a lack of coherence, unity or stability. A paradox-sensitive quality programme takes the same phenomena as an opportunity to foreground the question of form: not “How can we get rid of this heterogeneity?” but “What does it tell us about our observational form?”; not “How do we finally achieve consensus?” but “Which forms of observing are competing here?”; not “How do we secure evidence once and for all?” but “How do we design the recurrent return of our own uncertainty?”

Our proposal formulates a conditional internal claim: if science operates as a meaning- and code-bound system and its observations are tied to an invisibly maintained origin paradox, then quality increases where this paradox is addressed in the very course of operation in such a way that both the range of next possible connections and the robustness of the system increase. Quality would then no longer be the name for the state of a contradiction-free, well-ordered body of knowledge, but for the way in which a system handles its paradox: how it uses invisibilization to be able to decide, and visibility to be able to change. Coherence thereby does not lose all significance; it appears as a possible effect of addressed difference, not as a programme to erase difference.

## 6 Taking our own medicine: the paradox of paradox

The very shift from coherence to paradox already introduces a distinction—between coherence and paradox. Taken seriously, this means that a knowing system can no longer semantically frame quality primarily as the exclusion of contradiction but must treat it as dealing with the unity of the difference. The question is not what is true, but what is true and false—more precisely: under which forms true/false become selectable in each case. A coherence-focused paradigm favors “true or false”; a paradox-oriented perspective keeps “true and false” present as an operative unity, without leaving this guiding distinction.

Once this figure is bent back onto itself, the paradox of paradox becomes visible: research is coherent when it is both coherent and paradox; it fulfills a paradox-first orientation when it remains coherent while working through paradox. At the level of form, this is reflexivity in the strict sense: the application of the system's own basic operation to itself (truth-processing of truth-processing), with paradox functioning both as the topic and as the very mode of operation. As soon as the paradox is made visible, it withdraws from the very level of observation that made it visible: the re-entry briefly lifts the veil but consumes its own evidential force and shifts the paradox one level up. Visibility is therefore necessarily fleeting: the unity of the difference remains operative by becoming visible for a time and then invisible again. The effect is functional: autopoiesis is not blocked but gains elasticity in the face of perturbations, because the system carries its distinctions along as distinctions without becoming trapped in them. In short, the social dimension translates the paradox of observation into questions of recognition, authority and responsibility: who is allowed to keep a form invisible, and who is forced to account for it?

In systems-theoretical terms, this can be framed as a conditional internal claim: if science briefly indicates the paradox of its own observational forms in operation and does not erase it permanently, then its robustness in the sense of autopoiesis increases; the truth code remains stably applicable while the number of next possible operations grows. This concerns not only internal ordering (programmes, levels, temporal regimes) but also structural couplings: a reflexively formatted system processes external irritations as conditions for generating its own connections, without shifting its code into foreign logics. The gain does not appear as “more truth” as substance, but as more possibilities of connection within the truth process. In this light, Luhmann's call for a “re-thinking from ‘logical coherence' to paradox” (Luhmann, 1995a, p. 105) becomes readable as self-application: coherence is not discarded but rebound to the side of form. Coherence is then not a counter-principle to paradox but the effect of a practice that keeps the constitutive difference present to the right degree. Consensual integration—the smooth leveling of differences—is, under these conditions, not an ideal; it carries the risk of making paradoxes invisible to everyone and thereby conserving them for the time being. Keeping paradox visible is not an end in itself but the condition under which decisions can continue to be made without confusing their own premises. For science, paradox is thus not a problem to be eliminated but the (recognized or unrecognized) resource of its continuation. What changes under this perspective? Everything and nothing.

## 7 Epilog

The conceptual structure sketched here—form, origin paradox, proliferation, and a paradox-oriented understanding of quality—is not confined to science; science serves as a particularly transparent case of a knowing system that must live and operate with the paradox of its own observation.

With regard to the specific case of science, one may ask why the perspective developed here—although not new—has so far only selectively entered the scientific system. From a systems-theoretical point of view, the answer suggests itself: it regularly encounters quality and knowledge programmes that functionally invisibilize paradoxes in order to make decisions possible. A coherence-oriented paradigm filters out precisely what this perspective depends on—the briefly visible re-entry of its own distinction. A paradox-oriented paradigm of knowing presupposes, as its condition of possibility, that the form of observation is carried along in a dosed manner; within a programme that smooths out visibilities for the sake of stabilization, this necessarily appears as disturbance.

## Footnotes

1 The “first distinction” is understood from the perspective of systems-theory.
